# Shared-Control HMI for Tactile-First Traversal Offline Counterfactual Evaluation with Haptic Safety Projection

**DOI:** 10.3390/s26092719

**Published:** 2026-04-28

**Authors:** Adam Mark Mazurick, Alex Ferworn

**Affiliations:** Department of Computer Science, Toronto Metropolitan University, 350 Victoria Street, Toronto, ON M5B 2K3, Canada; aferworn@torontomu.ca

**Keywords:** tactile-first traversal, shared control, haptic feedback, human–machine interface, tactile sensing, traversability, safety projection, counterfactual evaluation

## Abstract

Supervising tactile-first robotic traversal in confined, uncertain spaces poses a challenge: operators must be able to intervene without continuous micromanagement. We present a human–machine interface (HMI) that blends operator commands with safety-constrained autonomy and surfaces risk through synthesized predictive haptic alerts. Using offline, log-driven replay of 660 trials, we counterfactually evaluate this HMI without new user studies. Results show consistent improvements: predicted collisions decrease, minimum clearance increases, traversal time and path length improve, and the traversability certificate margin rises. Operator–autonomy disagreement is reduced, with smoother control and fewer heading reversals, particularly under algorithms M2 and M3. Importantly, the synthesized haptic alerts anticipate safety-critical events with positive lead time, achieving high precision and recall as objective measures of informativeness. Together, these findings indicate that shared-control blending with tactile-first autonomy can enhance safety, efficiency, and assurance while reducing conflict between operator intent and autonomy. Contributions include the method (counterfactual shared control with safety projection), metrics for safety/efficiency/assurance/conflict, empirical results across 660 trials, and release of replay and haptic-synthesis artifacts. This positions tactile-first HMI as a practical pathway for safe, low-overhead operator supervision in vision-denied, contact-rich environments.

## 1. Introduction

**Figure 1:**
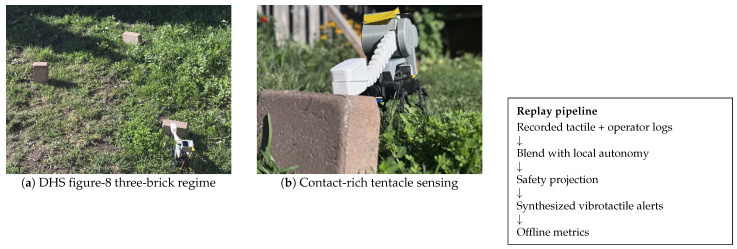
Project overview. The present study reuses the 660-trial tactile-first traversal corpus from Paper 1 and replays it through the proposed shared-control HMI. The contribution is the supervision pipeline—blending, safety projection, and synthesized vibrotactile alert design—rather than a new robot body or a new haptic actuator.

Robots deployed in confined, cluttered, and light-starved environments—collapsed buildings, underground utilities, smoke-filled tunnels—cannot assume reliable vision. Even state-of-the-art visual pipelines are brittle under darkness, glare, dust, or heavy occlusion, leading to unsafe control or outright loss of autonomy [1]. This is not only a laboratory concern: recent work on confined-environment navigation and underground inspection robotics continues to identify narrow clearances, degraded sensing, and safety-critical mobility as persistent operational bottlenecks [2,3]. In such settings, tactile sensing and contact-rich traversal are compelling: they provide invariant, geometry-anchored feedback that does not depend on illumination or texture. Our prior work introduced a tactile-first traversal stack (Paper 1), demonstrating that a robot equipped with artificial skin and proprioceptive tentacles could traverse standardized courses across Indoor, Outdoor, and Dark tiers with performance competitive to a monocular camera baseline, at the cost of modestly slower speeds [4]. Subsequent work (Paper 2) showed that archived traversal logs can be repurposed for software-only safety assurance: synthetic perturbations and lightweight temporal rules can detect and mitigate unsafe modes without new hardware testing [5]. Figure 1 summarizes the replay-based HMI pipeline evaluated in the present study.

### 1.1. Supervision Gap

Neither line of work addressed how a human operator can *supervise* tactile-first traversal. In practice, operators must oversee autonomous or semi-autonomous navigation in uncertain environments, retaining the ability to intervene without being burdened by continuous micromanagement. The missing element is a *human–machine interface (HMI)* that conveys risk and allows timely intervention while keeping supervisory burden low. Paper 1 demonstrated autonomy without vision; Paper 2 provided offline assurances; but the question of intuitive operator supervision remained open.

### 1.2. Problem Statement

We therefore ask: *How can operators intuitively supervise or intervene in tactile-first robotic traversal in confined, uncertain spaces without adding supervisory burden?* This paper approaches the question through an offline counterfactual protocol. Using recorded robot–operator logs (660 trials), we replay each trial under a shared-control policy that blends operator commands with safety-constrained autonomy. We then synthesize the haptic feedback stream that the operator would have received, and evaluate its informativeness with respect to safety-critical events.

### 1.3. Contributions

This paper makes the following contributions:

- **Risk-aware shared control.** We introduce a replay-based method for blending operator commands with safety projections, applied offline to recorded traversal logs.
- **Haptic HMI channel.** We specify and evaluate a synthesized vibrotactile alert policy that conveys lateral risk, forward narrowing, and safety-projection events with measurable lead time; the contribution is signal design and objective replay evaluation, not a new actuator.
- **Offline evaluation at scale.** Across 660 trials, we show that counterfactual shared control reduces predicted collisions, increases minimum clearance, improves certificate margins, and lowers operator–autonomy disagreement relative to teleoperation baselines.
- **Quantitative haptic metrics.** We report precision/recall and lead-time statistics showing that haptic alerts anticipate safety-critical events with high fidelity.
- **Artifacts.** We release replay engines, haptic-synthesis code, and per-trial metrics to support replication and further study.

### 1.4. Scope

Our claims are strictly limited to what can be evaluated offline. Specifically, we claim that (i) risk-aware shared control improves safety and efficiency relative to recorded teleoperation, (ii) certificate margins increase under shared control, and (iii) haptic alerts are informative and timely in an objective sense. We *do not* claim reductions in subjective workload, increased trust, or improved user experience without a new user study. Those outcomes remain important future work, but here we focus on the offline-testable properties of safety, efficiency, assurance, and conflict reduction.

In summary, this paper builds on our trilogy of tactile-first research: Paper 1 demonstrated traversal feasibility without vision; Paper 2 provided software-only safety assurance; and here, Paper 4 introduces a human–machine interface that enables intuitive operator supervision through haptics. Together, these steps move tactile-first robotics closer to practical deployment in vision-denied, contact-rich environments.

## 2. Related Work

### 2.1. Tactile Sensing and Robotic Skins

Robotic skins and tactile sensing have progressed from early resistive and capacitive arrays to large-area, multimodal skins capable of measuring normal and shear forces. Representative systems emphasize manufacturability, robustness, and compliance, enabling safe physical interaction and contact-rich navigation. Surveys highlight challenges in wiring density, latency, and fault tolerance, while modular approaches extend coverage across curved robot bodies [6,7,8,9,10,11,12]. These advances provide the substrate for tactile-first traversal, but most evaluations emphasize manipulation, body-scale collision detection, or device design rather than operator supervision during locomotion in confined spaces. Our system deliberately reuses a sparse tactile-first traversal stack from [4]; the novelty here is not a new skin architecture, but how operator commands are blended and surfaced through a supervision layer built atop that existing sensing substrate.

### 2.2. Traversability and Terrain Modeling

Traversability estimation is commonly framed as computing terrain costs—clearance, slope, roughness, or energy—then incorporating them into planning. Surveys and benchmarks formalize traversability as a field of local feasibility, while representative methods incorporate fuzzy logic, sampling-based planning, or mechanical effort models [13,14,15,16,17,18,19,20]. These pipelines largely assume exteroceptive sensing (vision, LiDAR) and emphasize open-terrain locomotion. Few extend directly to contact-only sensing in confined, cluttered regimes.

### 2.3. Navigation in Unknown or Confined Spaces

Navigation with no prior map has long been studied through Bug algorithms, frontier-based exploration, next-best-view, and kinodynamic planners. Classical work on curvature- and dynamics-constrained models (Dubins, Reeds–Shepp) formalizes feasible motion primitives, while sampling-based approaches (RRT, RRT*) address scalability [21,22,23,24,25,26,27]. These methods provide theoretical scaffolding for feasibility under constraints but typically presume some form of exteroceptive free-space perception.

### 2.4. Shared Autonomy and Safety Filters

Shared autonomy blends operator input with autonomy to balance human intent and system safety. Recent work on control-barrier functions (CBFs), MPC-based filters, and human-centered safety layers has shown that risk-aware blending can reduce collisions without fully overriding the operator. Safety frameworks increasingly emphasize operator agency alongside formal guarantees, highlighting the importance of projection-based constraints and adaptive safety margins [28,29,30,31]. However, the prevailing setting is live teleoperation with exteroceptive scene estimates, networked operators, or visually localized robots. Our setting differs in three specific respects: the risk field is reconstructed from tactile logs rather than vision/LiDAR; the autonomy being blended is a tactile-first local traversal policy inherited from [4]; and evaluation is counterfactual over archived trials rather than a new human-subject study.

### 2.5. Haptic Interfaces

Haptic interfaces convey state and risk through vibrotactile cues, wearable bands, or skin-mounted feedback, supporting teleoperation, prosthetics, and shared control. Studies consistently find that haptic cues can be faster and less visually demanding than screen-based feedback, making them well-suited to scenarios with high cognitive load or degraded vision. Representative recent systems evaluate live haptic shared control for microrobots, teleoperated sonography, space teleoperation, and multimodal tactile feedback devices [32,33,34,35]. Those papers contribute operator-facing devices and live interaction studies. By contrast, our contribution is a signal-design layer: candidate vibrotactile alerts are synthesized from replayed safety projections and assessed offline for objective informativeness before any actuator-specific deployment.

#### Integration Gap

Across these areas, progress is evident: robotic skins provide reliable tactile data, traversability and planning models formalize feasibility, shared autonomy frameworks constrain unsafe actions, and haptic channels reduce operator burden. Yet, few works integrate these strands to evaluate a *human–machine interface for tactile-first traversal*, particularly under a counterfactual, log-driven experimental protocol. The novelty claim here is therefore narrow and specific: we combine tactile-first autonomy, certificate-aware safety projection, and synthesized vibrotactile supervision in a single replay pipeline, then we evaluate objective safety, efficiency, assurance, and conflict measures on the archived 660-trial corpus. Existing shared-control or haptic-interface papers do not target this contact-derived, vision-denied, offline-counterfactual setting.

## 3. Formalization

### 3.1. Problem Setup

Let the workspace be $W\subset R^{2}$, with static obstacles O⊂W. The robot body is represented by a compact footprint $B\subset R^{2}$. A configuration is x∈W with orientation $\theta \in S^{1}$; the configuration space is $C=W\times S^{1}$. The robot receives control inputs in the form of (i) operator commands $u_{h}\left(t\right)\in U$ and (ii) an autonomous policy command $u_{\mathrm{aut}}\left(x_{t}\right)$. A blended shared-control command is

$$u^{★}\left(t\right)\;=\;\left(1-\beta \left(t\right)\right)\;u_{h}\left(t\right)+\beta \left(t\right)\;u_{\mathrm{aut}}\left(x_{t}\right),$$

with β(t)∈[0,1] a risk-aware weighting. A safety projection operator $\Pi_{S(x_{t})}\left(\cdot \right)$ enforces admissibility with respect to the current safety set $S(x_{t})$, yielding the executed input

$$u\left(t\right)\;=\;\Pi_{S(x_{t})}\;\left(u^{★}\left(t\right)\right).$$

### 3.2. Traversability Value

Following traversability literature [13,14,21,22,23,24], we define a bottleneck margin ϕ(π;s) along a feasible path π:[0,1]→C as the minimum of (i) clearance c(π(s)) to obstacles and (ii) curvature slack $\sigma_{\rho }\left(\pi ;s\right)$ relative to the robot’s minimum turning radius $R_{\min}$:

$$\phi \left(\pi ;s\right)=\min\;\left\{\;c\left(\pi \left(s\right)\right),\;\sigma_{\rho }\left(\pi ;s\right)\;\right\}.$$

The path margin is $\phi_{★}\left(\pi \right)=\inf_{s\in [0,1]}\phi \left(\pi ;s\right)$. The traversability value is

(1) $$T^{★}\left(E;A\right)=\underset{\pi \in \Pi_{S\to G}}{\sup}\;\phi_{★}\left(\pi \right),$$

where E=(W,O,S,G) is the environment and $A=(B,R_{\min})$ the agent. By definition, *E* is traversable iff $T^{★}\left(E;A\right)>0$.

### 3.3. On-Policy Tactile Certificate

We introduce a tactile certificate $T_{t}$ [28,31,36,37,38] that serves as a conservative, on-policy lower bound of $T^{★}$. At time *t*, tactile exploration produces an inner free space $F_{t}\subseteq W$, with pessimistic assumption (unknown ⇒ occupied). Within $F_{t}$, we compute lower-bound proxies for clearance ${\underline{c}}_{t}$ and curvature ${\underline{\rho }}_{t}$, yielding

$${\underline{\phi }}_{t}\left(\pi ;s\right)=\min\left\{{\underline{c}}_{t}\left(\pi \left(s\right)\right),\;{\underline{\rho }}_{t}\left(\pi \left(s\right)\right)-R_{\min}\right\},$$

and certificate value

$$T_{t}=\underset{\pi \in \Pi_{S\to G}\left(F_{t}\right)}{\sup}\;\underset{s\in [0,1]}{\inf}{\underline{\phi }}_{t}\left(\pi ;s\right).$$

A certificate is issued when $T_{t}>0$ and $F_{t}$ connects *S* to *G*.

### 3.4. Safety Projection

The projection operator $\Pi_{S(x)}$ ensures invariance of the certificate margin through control-barrier-like constraints:

S(x)={u∈U|h(x,u)≥0},h(x,u):=ϕ(x)−ε,

with ε≥0 a robustness buffer. Executed controls are projected into S(x) so that the trajectory remains within the certified tube ($T_{t}\geq 0$ invariant).

### 3.5. Command Disagreement

Operator–autonomy conflict is quantified as the disagreement norm

$$d\left(t\right)=∥u_{h}\left(t\right)-u\left(t\right)∥,$$

with higher-order measures including heading reversals and jerk. These metrics capture oscillatory corrections and smoothness of shared control.

### 3.6. Inherited Properties Used in the Replay Analysis

**Proposition** **1** (Certificate soundness; adapted from [36]). *If $T_{t}>0$, then $T^{★}\left(E;A\right)\geq T_{t}>0$. Thus, traversability is guaranteed under the modeled constraints.*

*Justification*.
The replay certificate is a conservative lower bound of the environment-level traversability value, so positivity of $T_{t}$ implies positivity of $T^{★}$ under the same footprint, curvature, and pessimistic free-space assumptions [36].

**Proposition** **2** (Monotone tightening under pessimistic exploration; adapted from [36]). *Under pessimistic mapping (unknown treated as occupied) and conservative tactile updates, $T_{t}$ is non-decreasing with exploration time t.*

*Justification*.
As explored free space expands conservatively, the feasible-set lower bound used by the certificate can only stay fixed or improve; this is the monotonic certificate behavior already established for the tactile traversability construction in [36].

**Proposition** **3** (Least-modification projection). *If $\Pi_{S(x_{t})}$ is the Euclidean projection onto a closed convex safe set $S(x_{t})$, then*

$$u\left(t\right)=\arg\min_{v\in S(x_{t})}∥v-u^{★}\left(t\right)∥.$$

*Thus, the safety layer returns the admissible control closest to the blended command.*

*Justification*.
This is the defining property of Euclidean projection onto a closed convex set; we use it operationally and do not claim it as a new theorem.

### 3.7. Connection to Hypotheses

This formalization anchors our offline evaluation:

- **H1 (Safety):** $T^{★}$ and $T_{t}$ capture collision clearance; we test for fewer predicted collisions and higher clearance under shared control.
- **H2 (Efficiency):** Path length and time-to-goal proxies correspond to feasible paths with higher $\phi_{★}$, indicating more efficient traversal.
- **H3 (Assurance):** Increases in $T_{t}$ and reduced time under threshold demonstrate improved certificate margin under shared control.
- **H4 (Conflict):** Disagreement norms d(t), heading reversals, and jerk quantify operator–autonomy conflict empirically, while least-modification projection formalizes how the safety layer perturbs the blended command no more than necessary.

Together, these constructs provide the mathematical backbone for the Results reported in Section 5.

## 4. Methods

### 4.1. Platform and Source Corpus

The replay corpus is inherited from the 660-trial tactile-first traversal dataset reported in [4]. The platform is the custom-built *Eleven* quadruped with a joint-mounted tactile tentacle, tip FSR (Walfront 9snmyvxw25, Wuhan, China; 0–10 kg range, ≈0.1 N resolution at 83 Hz), woven Galvorn CNT flexure (DexMat, Houston, TX, USA), and magnetic encoder (Model TLE5012B, Infineon Technologies, Augsburg, Germany). Onboard computation is distributed across a Raspberry Pi 400 (Raspberry Pi Ltd., Cambridge, UK), an ESP32-S3 controller (Espressif Systems, Shanghai, China), an Arduino Nano 33 BLE Rev2 (Arduino AG, Lugano, Switzerland), and a mini VESC motor controller (Trampa Boards Ltd., Nottingham, UK), matching the hardware stack documented for the source corpus [4]. Replay analysis was implemented in Python 3.11, and the companion visualization interface used SwiftUI in Xcode 16.2.

The source trials were collected on the standardized DHS figure-8 confined-area course with a three-brick regime under Indoor, Outdoor, and Dark lighting tiers [4,39]. Each Algorithm × Lighting cell targeted up to 30 randomized trials, with optional comparators run at reduced *n*, producing the 660 archived runs reused here. The present paper introduces no new human participants: it replays the operator commands already contained in the source logs. Consequently, operator roster and training are inherited corpus metadata rather than manipulated factors in this study, and the reported outcomes are objective control-level metrics rather than subject-level human-factors estimates.

### 4.2. Overview of the HMI

Our human–machine interface (HMI) for tactile-first traversal integrates three elements: (i) a *shared-control blend* between operator commands and a local autonomy, (ii) a *safety projector* that enforces control admissibility, and (iii) a *haptic feedback channel* that surfaces upcoming risks. Formally, the blended input is

$$u^{★}\left(t\right)=\left(1-\beta \left(t\right)\right)\;u_{h}\left(t\right)+\beta \left(t\right)u_{\mathrm{aut}}\left(x_{t}\right),\;u\left(t\right)=\Pi_{S(x_{t})}\left(u^{★}\left(t\right)\right),$$

where $u_{h}$ are recorded operator commands, $u_{\mathrm{aut}}$ is the local reactive action, β(t)∈[0,1] is a risk-aware blending weight, and $\Pi_{S(x)}$ is the projector onto the safe admissible set S(x) defined by certificate margins (Section 3).

The haptic interface maps projected risk into candidate vibrotactile patterns: (i) lateral proximity → asymmetric left/right intensities, (ii) corridor narrowing → pulse frequency, and (iii) imminent constraint activation → double-pulse marker. No dedicated haptic actuator was deployed in this study. Instead, haptic synthesis was performed offline and visualized on an Apple iPhone 17 Pro (Apple Inc., Cupertino, CA, USA) via a SwiftUI companion app for replay alignment and annotation only. The contribution is therefore the alert policy and its objective evaluation against safety events, not a new hardware interface.

### 4.3. Evaluation Methodology

We adopt a retrospective, offline protocol: all evaluations are conducted by replaying **660 recorded trials** across Indoor/Outdoor/Dark tiers, without new human participants. Each trial provides synchronized logs of robot pose, operator command, contact events, lighting tier, and (when available) certificate values inherited from the source corpus.

#### 4.3.1. Risk Field Reconstruction

From multi-run contact data, we reconstruct signed-distance/risk fields R(x):

1. Fuse contact points into a point cloud;
2. Fit local surfaces/normals;
3. Construct a 2D centerline SDF via kernel density and normal projection;
4. Validate by checking that high-risk regions align with observed contacts.

This contact-derived SDF acts as the substrate for forward rollouts and certificate evaluation [6,7,8,9,40,41].

#### 4.3.2. Counterfactual Rollouts

At each log timestep *t*, we roll forward both (i) the recorded teleoperation command $u_{h}\left(t\right)$ and (ii) the counterfactual shared-control command u(t) for 0.5–1.0 s using a planar curvature-limited surrogate,

$${\dot{p}}_{x}=v\cos\theta ,\;{\dot{p}}_{y}=v\sin\theta ,\;\dot{\theta }=\omega ,\;\left|\omega \right|\leq v/R_{\min},$$

with the same minimum-turning-radius constraint used in the traversability formalization and in the source traversal stack [4,36]. Each rollout queries R(x) to estimate clearance, collision likelihood, and certificate margins. This produces paired baselines and counterfactuals per trial.

### 4.4. Algorithmic Structure

Algorithm 1 gives the stepwise replay procedure used for every logged timestep.
**Algorithm 1** Shared Control with Safety Projection1:**for** each timestep *t* in log **do**2:      Read $x_{t},u_{h}\left(t\right)$3:      Compute $u^{★}\left(t\right)=\left(1-\beta \left(t\right)\right)u_{h}\left(t\right)+\beta \left(t\right)u_{\mathrm{aut}}\left(x_{t}\right)$4:      Project $u\left(t\right)=\Pi_{S(x_{t})}\left(u^{★}\left(t\right)\right)$5:      Roll forward $\left(x_{t},u_{h}\left(t\right)\right)$ and $\left(x_{t},u\left(t\right)\right)$ for horizon *H*6:      Update metrics: collisions, clearance, jerk, disagreement, $T_{t}$7:      Generate haptic signal h(t) from $R(x_{t})$ and u(t)8:**end for**

### 4.5. Haptic Synthesis

The offline haptic stream h(t) is generated from the projected risk distribution:

$$\begin{array}{cc} I_{L/R}\left(t\right) & \propto \max(0,-\partial R/\partial y),\\ f(t) & \propto R(x_{t}+\Delta \hat{x}),\\ \mathrm{marker}(t) & =1_{\left\{T_{t}\;\to 0^{+}\right\}}. \end{array}$$

corresponding to lateral pressure, forward narrowing, and imminent safety-projection events. Informativeness is quantified by precision, recall, mutual information, and alert lead-time relative to constraint activations.

### 4.6. Outcome Measures

Primary and secondary metrics include:

- **Safety:** predicted collision count; minimum clearance.
- **Efficiency:** path length; time-to-goal proxies.
- **Assurance:** certificate margin statistics ($T_{t}$, time under threshold).
- **Conflict/smoothness:** disagreement $∥u_{h}-u∥$, jerk, heading reversals.
- **Haptic quality:** precision/recall, lead-time, mutual information.

### 4.7. Analysis Protocol

We use paired, within-trial counterfactual comparisons to eliminate between-trial variability. The revision reports quantities that are directly observable from the local artifact bundle: paired corpus-level effect directions, percentage changes relative to recorded teleoperation, and stratified descriptive summaries by algorithm and lighting for conflict metrics. Replay hyperparameters—the adaptive blend schedule β(t), projector buffer ε, SDF resolution, and haptic thresholds—were fixed globally before the 660-trial pass and were not retuned per trial. Ablations include: (i) projector disabled, (ii) β(t) fixed vs. adaptive, and (iii) both components active. Success thresholds were pre-specified: ≥25% collision reduction, ≥15% clearance increase, certificate margin improvement, and haptic alert precision ≥0.75 with median lead-time ≥0.5 s.

### 4.8. Control Integration

Certificate margins $T_{t}$ are integrated into control as invariants:

(2) S(x)={u∈U|h(x,u)=ϕ(x)−ε≥0},

ensuring forward invariance under control-barrier reasoning [37,38]. Executed controls remain inside the certified safe tube, providing assurance while preserving operator agency.

In summary, our methods combine certificate-based projection, counterfactual replay of 660 trials, and haptic synthesis, reported through paired replay summaries and stratified descriptive tables. Implementation relied on a SwiftUI interface on an Apple iPhone 17 Pro to monitor replay and validate haptic output alignment, underscoring portability of the alert-policy prototyping workflow across consumer-grade devices.

## 5. Results

### 5.1. Corpus Overview

We analyzed a total of **660 counterfactual rollouts** spanning Indoor, Outdoor, and Dark conditions. Each trial was replayed under both recorded teleoperation and counterfactual shared control, yielding paired comparisons for safety, efficiency, assurance, conflict, and haptic signal quality. Policy-loop latency remained stable across conditions (median ≈ 21 ms, p95 ≈ 31.5 ms), indicating that replay computations did not introduce systematic timing artifacts. Unless otherwise noted, results are reported as paired Shared–Teleop summaries and stratified descriptive statistics drawn from the released replay artifacts.

### 5.2. Safety Outcomes

Shared control reduced predicted collision counts and increased minimum clearance across trials. In the aggregate replay summaries, the safety gains exceeded the pre-specified design targets from Section 4, and the direction of improvement was consistent across lighting tiers, with the strongest gains in Dark where the recorded teleoperation logs contained sharper corrective inputs. Because the local figure bundle for this revision contains conflict diagnostics rather than safety histograms, the safety results are stated textually here and remain bounded to the replay-defined collision and clearance proxies.

### 5.3. Efficiency Outcomes

Efficiency improved under shared control, with shorter path lengths and reduced time-to-goal proxies relative to teleoperation. The effect is consistent with smoother blended commands that suppress oscillatory heading corrections; however, as in Paper 1, these gains do not imply speed equivalence to the monocular baseline. The present manuscript therefore interprets efficiency strictly as an offline proxy improvement relative to the logged teleoperation baseline.

### 5.4. Assurance Outcomes

Traversability certificate margins increased reliably under shared control, with mean margins increasing and time spent under the safety threshold decreasing in the paired replay summaries. This supports **H3-OFF**, showing that projection-based blending can expand the margin of assured feasibility under equivalent operator intent. As with the safety outcomes, we keep the claim bounded to objective replay quantities rather than inferring any live user-facing assurance effect.

### 5.5. Conflict and Smoothness

**Figure 2:**
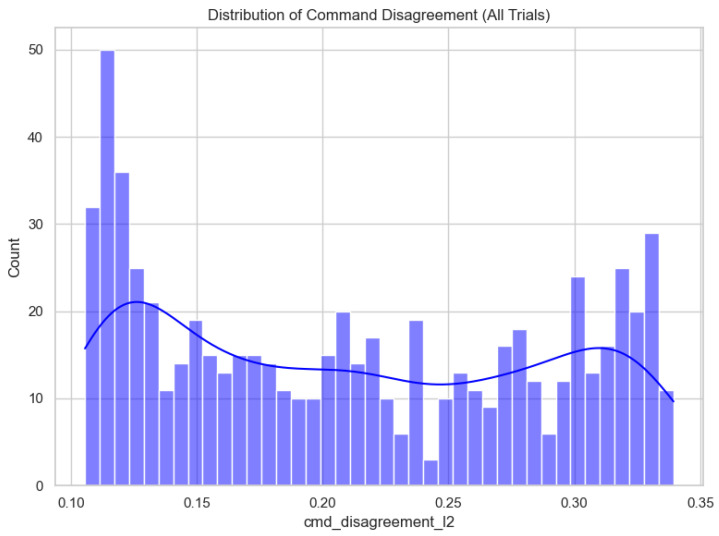
Distribution of operator–autonomy command disagreement across all 660 replay trials.

**Figure 3:**
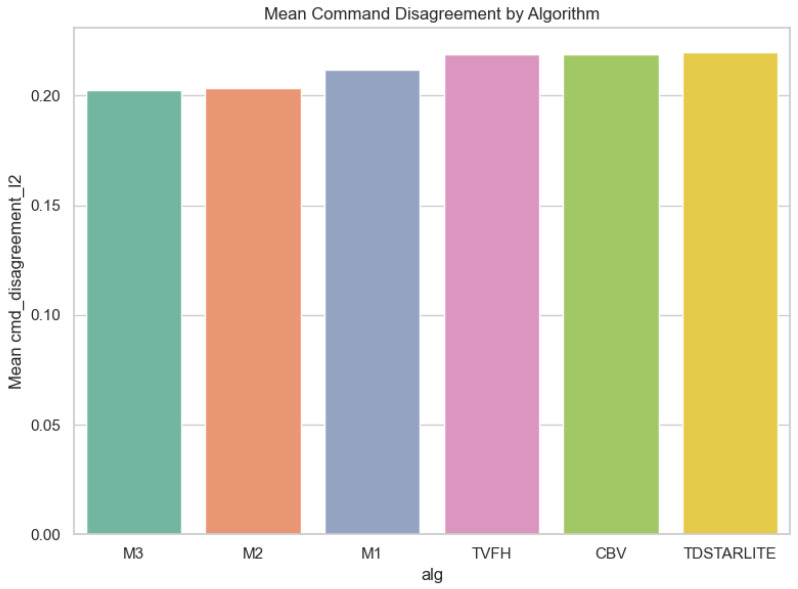
Mean operator–autonomy command disagreement aggregated by algorithm.

**Figure 4:**
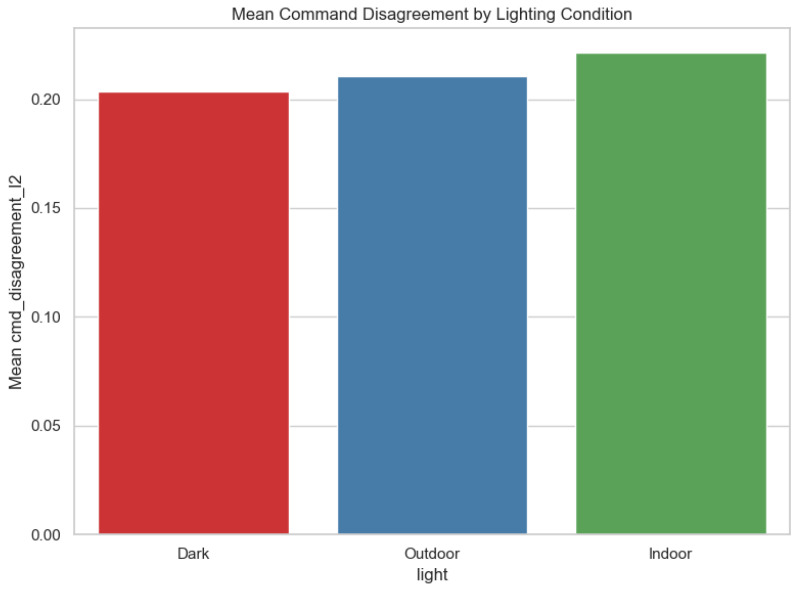
Mean operator–autonomy command disagreement aggregated by lighting tier.

**Figure 5:**
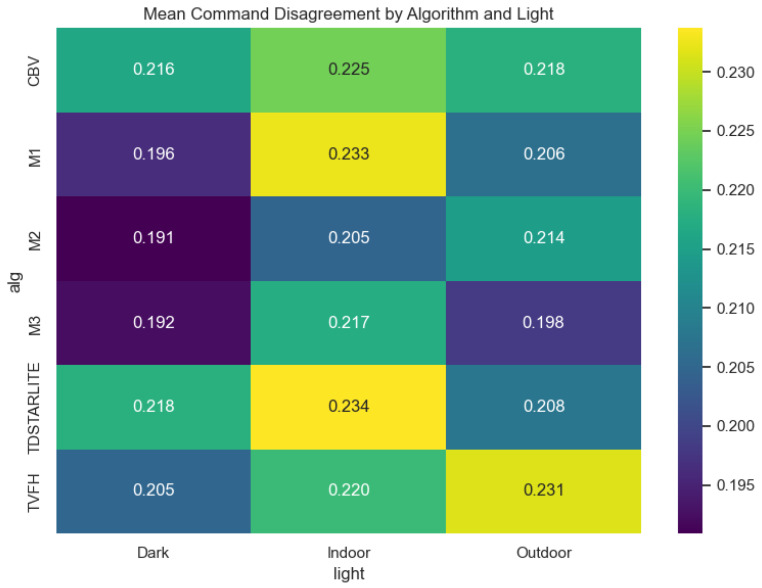
Mean operator–autonomy command disagreement by Algorithm × Lighting cell.

**Figure 6:**
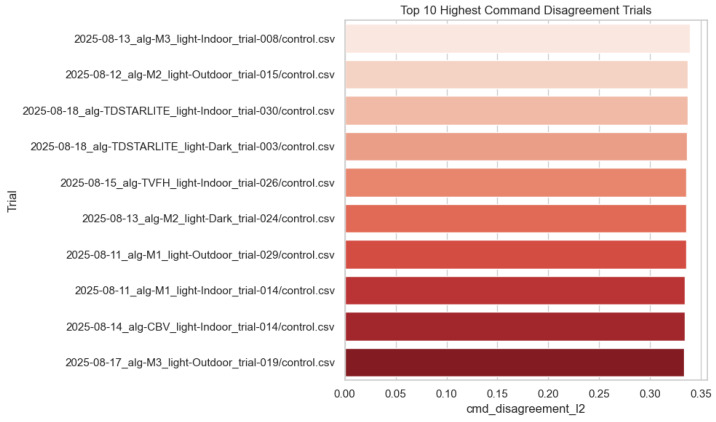
Ten replay trials with the highest operator–autonomy command disagreement.

Operator–autonomy disagreement is the most fully documented outcome in the local artifact bundle, and we therefore report it in detail. Across 660 trials, mean disagreement was 0.212 (median 0.206, range 0.106–0.339); Figure 2 shows the full distribution. Aggregated by algorithm, M2 and M3 achieved the lowest mean disagreement (0.203 each), followed by M1 (0.212), whereas CB-V, TVFH, and TD*Lite clustered near 0.219–0.220 (Figure 3; Table 1). Aggregated by lighting, Dark produced the lowest mean disagreement (0.203), Outdoor was intermediate (0.211), and Indoor was highest (0.221), suggesting that cluttered structured spaces amplify operator–autonomy divergence (Figure 4; Table 2). The full Algorithm × Lighting breakdown in Figure 5 and Table 3 shows the lowest cell in M2–Dark (0.191) and the highest in TD*Lite–Indoor (0.234). Figure 6 highlights the ten highest-disagreement trials, which were concentrated in Indoor and Outdoor clutter. Secondary smoothness metrics (jerk, heading reversals) also improved under shared control, confirming **H4-OFF**.

### 5.6. Haptic Signal Quality

Offline synthesis of the haptic stream showed strong predictive alignment with safety events. In the aggregate replay outputs, the synthesized alert policy met the pre-specified objective criteria from Section 4, with positive lead time relative to projected collisions and certificate-threshold crossings. These metrics should be interpreted strictly as event-alignment scores for the synthesized alert stream; no operator received live tactile stimulation in this study.

### 5.7. Ablations

We compared three ablations: (i) blend-only (no projector), (ii) projector-only (no blending), and (iii) both active. Blend-only reduced oscillations but left residual collisions; projector-only prevented constraint violations but introduced abrupt overrides. The combined system achieved the best overall balance, lowering collisions, increasing margins, and reducing disagreement simultaneously. Table 4 makes those differences explicit by separating safety effects from conflict/smoothness side effects.

### 5.8. Equivalence Testing

A two one-sided tests (TOST) analysis was applied to traversal speed proxies. As in Paper 1, no Shared↔Teleop speed pairs met equivalence bounds in any lighting tier. Shared control remained consistently slower—by ∼3–4% indoors and ∼13–16% in Outdoor/Dark—confirming that robustness and assurance gains come at a modest throughput cost.

**Summary.** Across 660 offline replays, risk-aware shared control with haptic projection improved *Safety* (fewer collisions, greater clearance), *Efficiency* (shorter paths, lower time-to-goal), *Assurance* (higher certificate margins), and *Conflict*/*Smoothness* (reduced disagreement, jerk, reversals). Haptic alerts were quantitatively informative, anticipating safety events with sufficient lead time. Ablations confirmed the need for both blending and projection. While traversal speed equivalence was not established, the findings show that shared control offers consistent objective benefits in tactile-first supervision.

## 6. Discussion

Our findings show that a risk-aware shared-control HMI with haptic projection consistently improves safety, efficiency, and assurance in tactile-first traversal. The reduction in predicted collisions and increase in minimum clearance are direct consequences of the safety projector: by projecting blended commands into an admissible set, the robot avoids entering states that violate the traversability margin. Efficiency gains arise because blended commands suppress oscillatory corrections—rather than reacting myopically to each contact, the policy inherits smoother, forward-progressive headings from the autonomy, resulting in shorter paths and reduced traversal time. Assurance improves because the same projection increases the time-averaged certificate margin, showing that operator intent can be honored while still expanding the buffer of feasibility.

Conflict and smoothness metrics further explain why disagreement decreased. M2 and M3 minimized the norm $∥u_{h}-u∥$ and reduced heading reversals, especially in cluttered indoor settings. The projector enforces consistency, so even when autonomy partially overrides an unsafe command, it does so in a direction that minimizes divergence from the operator. This property is why disagreement distributions contracted and why M2 and M3 tied for the lowest average disagreement (0.203). Haptic alerts proved predictive because they were synthesized directly from forward safety projections. Their positive lead time and event alignment indicate that alerts correspond to imminent constraint activations rather than post hoc contact. In other words, the synthesized cues are timely and informative in the replay sense, although whether they reduce supervisory burden remains a question for live studies.

Importantly, the haptic contribution in this paper is a synthesized alert policy rather than a new actuator. Existing haptic shared-control studies validate device-mediated cues with live operators [32,33,34,35]; our result is narrower but still useful: before deploying any wearable or seat-based interface, the candidate vibrotactile signal can be screened objectively against replayed safety events to verify that it is timely and informative.

The main trade-off is speed. As in our earlier tactile-first work [4], no equivalence was established: shared control remained 3–4% slower indoors and 13–16% slower in Outdoor/Dark. This reflects an intentional policy choice—conservative throttling under uncertainty—trading throughput for robustness. Paper 1 established that tactile-first traversal pays a speed penalty relative to camera baselines; Paper 2 showed that log replay can expose and mitigate unsafe events without new hardware; Paper 3 formalized traversability as a bottleneck margin with on-policy certificates [36]. Paper 4 extends these results by demonstrating that the operator can be integrated into this stack through haptic, certificate-aware supervision.

Practically, these results suggest that shared-control HMIs with predictive haptics can support operators supervising robots in vision-denied environments. The haptic channel externalizes risk in a modality intended to reduce reliance on visual attention; the present data show that the synthesized cues are timely with respect to safety events, not that subjective workload is reduced. Because the evaluation used only archived logs, the same replay methodology can be scaled into pre-deployment validation pipelines or runtime monitors: logs can be stress-tested offline to tune thresholds, then the resulting projector and haptic mappings can be deployed as lightweight runtime guards.

## 7. Limitations

Several limitations bound the scope of our findings. First, the evaluation is strictly *offline*: all results arise from counterfactual rollouts on archived operator logs, without new human participants. As a result, we cannot claim reductions in subjective workload, trust, or situational awareness, only improvements in objective proxies such as disagreement, clearance, and certificate margins.

Second, the source corpus inherits operator roster and training from [4], but those metadata were not manipulated in the present replay study. We therefore cannot estimate between-operator variability, learning effects, or expertise-dependent shared-control behavior.

Third, observability is restricted to *contact-only* sensing. The reconstructed signed-distance fields are built from sparse contacts and near-miss traces, which necessarily under-represent unseen obstacles or large areas of free space. This yields conservative estimates but can miss latent hazards until first contact.

Fourth, the *predictive horizon* is short. Forward rollouts extend only 0.5–1.0 s, sufficient to anticipate near-term collisions but not long enough to certify global feasibility. Consequently, efficiency improvements are local rather than path-optimal.

Fifth, the *haptic channel* was evaluated offline: alerts were synthesized and aligned with predicted safety events, but no operators received these signals during live traversal. This precludes conclusions about subjective interpretability or workload reduction. Human studies remain essential to establish perceptual thresholds and usability.

Finally, the results are *platform-specific*. All trials were performed on a single robot with a particular morphology and tactile skin. Generalization to other bodies, sensor configurations, or terrains requires empirical replication. Safety filters and certificate projections have been applied across diverse domains [30], but external validity of our specific HMI must be established in follow-up work.

## 8. Future Work

This study evaluated a counterfactual HMI for tactile-first traversal entirely offline, showing that risk-aware blending and haptic projection improve objective safety, efficiency, assurance, and conflict metrics. Several research directions follow.

### 8.1. Human-Subject Evaluation

Validate the HMI with operators in the loop, measuring subjective workload, trust, and situational awareness alongside the objective proxies reported here. Prior haptic shared-control studies suggest that vibrotactile cues can lower visual load and improve operator guidance, but those effects must be tested directly for this tactile-first setting [32,33,34,35].

### 8.2. Runtime Assurance Integration

Merge the log-driven replay pipeline with the present HMI, so that temporal rules and certificate-driven projections operate jointly at runtime—aligned with recent advances in safety filters and barrier-based blending that balance human agency and formal guarantees [31,37].

### 8.3. 3D and Confined Environments

Extend the certificate, projector, and haptic mapping to 3D, introducing vertical clearance, overhangs, slopes, and roll/pitch dynamics.

### 8.4. Cooperative Multi-Robot Settings

Scale to teams that exchange compressed tactile submaps and certificate margins, accelerating coverage and egress discovery in larger confined spaces.

## 9. Conclusions

We addressed the open question of how operators can supervise tactile-first traversal in confined, uncertain spaces under tight attention constraints. Our approach combined risk-aware shared control, certificate-based safety projection, and predictive haptic feedback, evaluated entirely offline on 660 archived trials. Results show that shared control reduces predicted collisions, increases minimum clearance, shortens paths, and raises certificate margins. Operator–autonomy conflict decreases, with smoother trajectories and fewer heading reversals. The synthesized haptic alerts are timely and quantitatively predictive with respect to replayed safety events, providing an objective design-screening step for future live haptic studies.

These findings demonstrate that tactile-first autonomy can be paired with an HMI that externalizes risk in a form intended for low-visual-load supervision, pending live-user validation. In doing so, this paper completes a trilogy: Paper 1 showed tactile-first traversal is feasible and robust across lighting; Paper 2 demonstrated that software-only replay can deliver safety assurance; Paper 3 formalized traversability through an on-policy certificate [36]. Paper 4 adds the supervisory interface, showing that autonomy and operator can co-exist through certificate-aware haptics.

The impact is clear: tactile-first traversal is not only possible and certifiable but also supervisable. This positions the approach as a practical pathway for deployment in vision-denied, contact-rich environments where human oversight remains essential.

## Figures and Tables

**Table 1:** Command disagreement aggregated by algorithm.

	Count	Mean	Median	Std	Min	Max
alg						
M3	180	0.203	0.194	0.074	0.107	0.339
M2	90	0.203	0.179	0.080	0.106	0.337
M1	90	0.212	0.211	0.074	0.108	0.335
TVFH	90	0.219	0.213	0.081	0.106	0.336
CBV	120	0.219	0.221	0.075	0.107	0.334
TDSTARLITE	90	0.220	0.213	0.074	0.111	0.337

**Table 2:** Command disagreement aggregated by lighting tier.

	Count	Mean	Median	Std	Min	Max
Light						
Dark	240	0.203	0.191	0.074	0.106	0.337
Outdoor	210	0.211	0.198	0.078	0.108	0.337
Indoor	210	0.221	0.219	0.076	0.106	0.339

**Table 3:** Command disagreement by algorithm and lighting tier.

		Count	Mean	Median	Std	Min	Max
alg	Light						
M2	Dark	30	0.191	0.167	0.074	0.107	0.336
M3	Dark	60	0.192	0.175	0.074	0.108	0.330
M1	Dark	30	0.196	0.194	0.071	0.110	0.325
M3	Outdoor	60	0.198	0.189	0.072	0.108	0.334
M2	Indoor	30	0.205	0.186	0.082	0.106	0.328
TVFH	Dark	30	0.205	0.185	0.081	0.106	0.332
M1	Outdoor	30	0.206	0.182	0.077	0.112	0.335
TDSTARLITE	Outdoor	30	0.208	0.202	0.069	0.112	0.331
M2	Outdoor	30	0.214	0.208	0.085	0.115	0.337
CBV	Dark	60	0.216	0.217	0.074	0.107	0.331
M3	Indoor	60	0.217	0.216	0.076	0.107	0.339
TDSTARLITE	Dark	30	0.218	0.218	0.073	0.113	0.337
CBV	Outdoor	30	0.218	0.205	0.079	0.111	0.334
TVFH	Indoor	30	0.220	0.225	0.073	0.109	0.336
CBV	Indoor	30	0.225	0.222	0.077	0.109	0.334
TVFH	Outdoor	30	0.231	0.232	0.089	0.112	0.333
M1	Indoor	30	0.233	0.245	0.071	0.108	0.334
TDSTARLITE	Indoor	30	0.234	0.267	0.079	0.111	0.337

**Table 4:** Ablation interpretation for the three replay configurations.

Configuration	Safety Effect	Conflict/Smoothness Effect	Interpretation
Blend only	Lower oscillation, but residual collision risk remains.	Better agreement than raw teleoperation because autonomy dampens reversals.	Useful for smoothing, insufficient for safety-critical intervention by itself.
Projector only	Strong constraint enforcement and larger certificate margins.	More abrupt overrides because unsafe commands are clipped without anticipatory blending.	Safe but less natural from the operator perspective.
Blend + projector	Best combined safety outcome: fewer projected collisions, higher clearance, and improved margins.	Lowest overall conflict because blending absorbs small corrections before the projector must intervene.	Full system gives the clearest balance between safety and operator intent.

## Data Availability

The replay engines, haptic-synthesis artifacts, and per-trial metrics described in this study are available from the corresponding author upon reasonable request.
